# Modelling the dynamics of EBV transmission to inform a vaccine target product profile and future vaccination strategy

**DOI:** 10.1038/s41598-019-45381-y

**Published:** 2019-06-26

**Authors:** Lara Goscé, Joanne R. Winter, Graham S. Taylor, Joanna E. A. Lewis, Helen R. Stagg

**Affiliations:** 1 University College London, Institute for Global Health, London, WC1N 1EH UK; 2 University of Birmingham, Institute of Immunology and Immunotherapy, Birmingham, B15 2TT UK; 3 MRC Centre for Global Infectious Disease Analysis, Department of Infectious Disease Epidemiology, Imperial College, School of Public Health, London, W2 1NY UK; 4 Usher Institute of Population Health Sciences and Informatics, The University of Edinburgh, Edinburgh, UK

**Keywords:** Viral infection, Cancer epidemiology, Applied mathematics

## Abstract

Epstein-Barr virus (EBV) is one of the most common human viruses and the cause of pathologies such as infectious mononucleosis (IM) and certain cancers. No vaccine against EBV infection currently exists, but such vaccines are in development. Knowledge of how EBV is transmitted at the population level is critical to the development of target product profiles (TPPs) for such vaccines and future vaccination strategies. We present the first mathematical model of EBV transmission, parameterised using data from England, and use it to compare hypothetical prophylactic vaccines with different characteristics and the impact of vaccinating different age groups. We found that vaccine duration had more impact than vaccine efficacy on modelled EBV and IM prevalence. The age group vaccinated also had an important effect: vaccinating at a younger age led to a greater reduction in seroprevalence but an increase in IM cases associated with delayed infection. Vaccination had impact on cancer incidence only in the long run, because in England most EBV-related cancers arise in later life. Durability of protection should be a key factor to prioritise in EBV vaccine development and included in vaccine TPPs. These findings are timely and important for vaccine developers and policy-makers alike.

## Introduction

Epstein-Barr virus (EBV) is a gamma herpes virus and one of the most common human viruses; it is estimated that about 90% of the world’s adult population is infected^1,2^. Infection usually occurs in childhood, this occurs earlier in poorer settings, and it is often delayed until adolescence in developed countries^3,4^. Transmission is through saliva^5^ and infection is usually asymptomatic, but in some instances it may cause infectious mononucleosis (IM) or particular cancers such as Hodgkin’s lymphoma, Burkitt’s lymphoma, gastric cancer, nasopharyngeal carcinoma and diffuse large B cell lymphomas^6,7^. IM increases the risk of developing Hodgkin’s lymphoma in the ten year period after infection^8^. Immunocompromised individuals are at an increased risk of developing EBV-associated disease^9^.

It has been estimated that an EBV vaccine could prevent nearly 200,000 new malignancies each year, worldwide^10^. EBV infection-preventing vaccines are currently in development^10,11^; a second generation EBV vaccine candidate is about to enter first-in-human trials^12^ and interest in the area continues to increase^13^. Consequently we have a window of opportunity to influence vaccine research and development. Target product profiles (TPPs) are planning tools that delineate a requisite set of attributes for new products to ensure that they are correctly profiled for the needs of the environment in which they will operate. For example, the financial viability of a vaccine candidate at the population level depends upon how key vaccine characteristics such as efficacy and duration of protection affect both EBV transmission and disease progression, and how this varies in different settings. A perfect or near-perfect vaccine is highly unlikely - the only herpesviral vaccine currently licensed, VZV oka, is only 85% effective when giving a single dose^14^- therefore there is a need a for a TPP specifying the characteristics of a potentially useful vaccine, and for the modelling tools to develop one.

In this work we develop a simple compartmental model^15^ of EBV transmission and use it to test several hypothetical vaccines, varying the efficacy, duration of protection and age group targeted for vaccination. No dynamical model has ever previously been created to study the spread of EBV and the sequelae of infection. Our model is calibrated using data from England, but can easily be parameterised to other settings, making it globally informative.

## Methods

### Model

**Figure 1:**
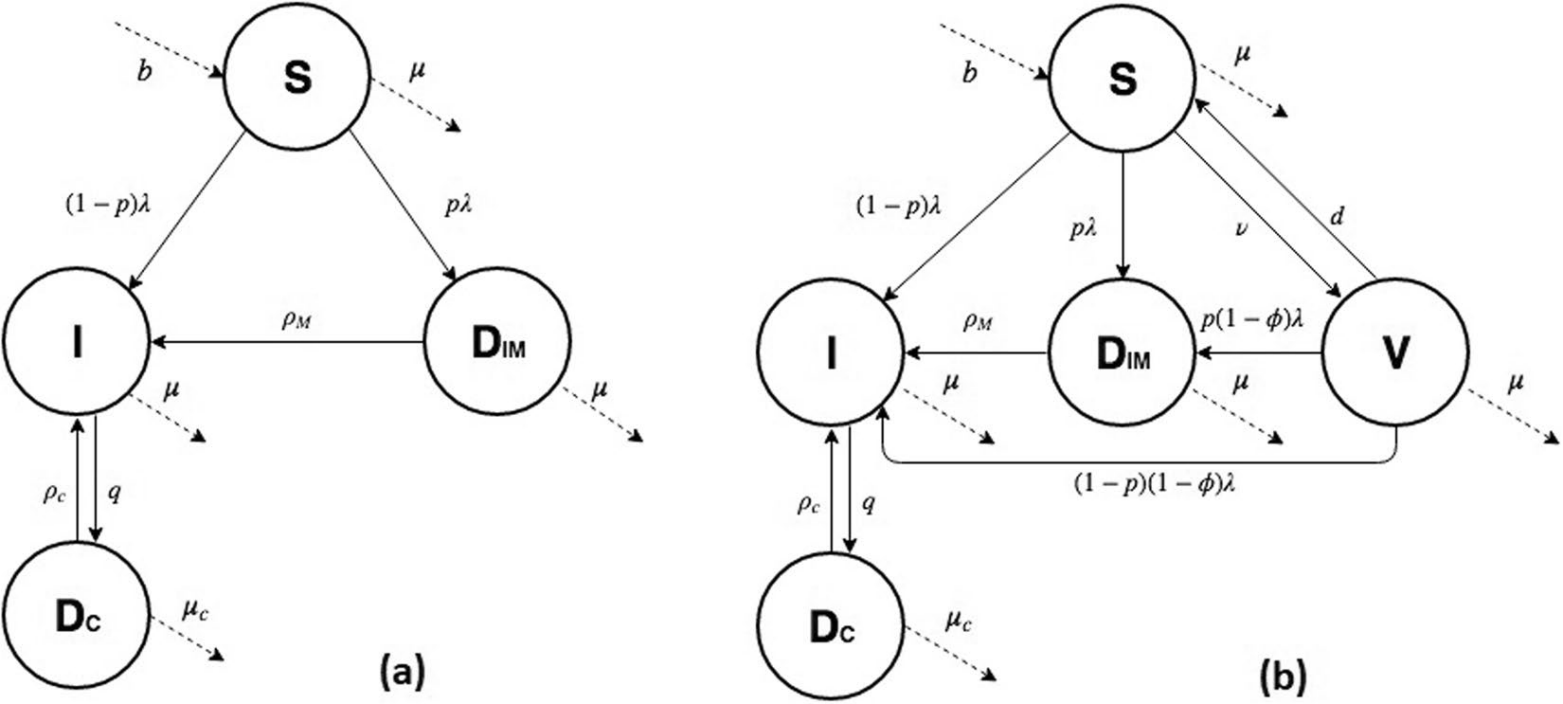
(**a**) EBV model: Schematic describing the spread of EBV in a population. All individuals are born susceptible ***S*** at rate *b*. They become infected with force of infection *λ*. Once infected, they have a probability *p* of developing IM and move to the IM compartment ***D***_*IM*_, where they remain until they recover, at rate *ρ*_*M*_. Post-IM, individuals move to the EBV-infected compartment ***I***. Infected individuals who do not develop IM also sit here. Unless a person develops cancer and moves to compartment ***D***_*C*_ at rate *q*, they remain in ***I*** for life. From ***D***_*C*_ people can either die, at death rate *μ*_*c*_ which is higher than the natural death rate *μ*, or they can recover at rate *ρ*_*c*_ and move back to compartment ***I***. (**b**) EBV model with vaccination: Schematic describing the spread of EBV in a population, including the possibility of vaccination. All individuals are born susceptible ***S***. A fraction of them are vaccinated pre-infection at a rate *ν* and move to the vaccinated compartment ***V*** where they may nonetheless be infected with a force of infection *λ* which is reduced according to the vaccine efficacy *ϕ*. Vaccine protection is lost with time, and individuals move back to the susceptible compartment at rate *d*.

We created a compartmental model (Fig. 1) for the spread of EBV, described using ordinary differential equations (Appendix). The model is calibrated to data from England but, given its simple structure, can easily be parameterised to study EBV transmission in other settings.

In our model, the population is divided into a single susceptible compartment ***S*** and three infected compartments: asymptomatic-EBV infected ***I***, IM cases ***D***_*IM*_ and cancer cases ***D***_***C***_ (Fig. 1a). All individuals are born susceptible to the infection, entering compartment ***S***. Susceptible individuals become infected at a rate that depends on the rate of contact with infectious individuals and the per-contact transmission probability, both of which are age dependent. When susceptible individuals become infected, they have a probability *p* of developing IM and moving to the IM compartment ***D***_*IM*_. Otherwise, they move to the asymptomatic EBV-infected compartment ***I***. Individuals recovering from IM (but not from EBV infection) also flow into compartment ***I***. EBV infection is lifelong and cannot be cleared immunologically or by treatment, so infected people remain in compartment ***I***. From here they may develop one of the most common EBV-associated cancers: Hodgkin’s lymphoma, Burkitt’s lymphoma, gastric cancer or nasopharyngeal carcinoma, and move to compartment ***D***_***C***_. Individuals can leave this compartment through death or recovery; in the latter case, individuals move back to the EBV-infected compartment ***I***. For simplicity, all cancers are included in a single compartment and IM is not considered as a possible risk factor for cancer. All individuals, at any age and any stage of infection, are affected by natural mortality. Age heterogeneity was included in the model by dividing the population into age groups, within which individuals’ ages were uniformly distributed.

To test possible vaccination strategies, we modified the model by adding an extra compartment for vaccinated individuals (Fig. 1b). Susceptible individuals are vaccinated at a rate *ν* and move to the vaccinated compartment ***V***. All uninfected individuals who are not protected by vaccination remain in the susceptible compartment ***S***. Vaccinated individuals can still be infected and develop IM, depending on the efficacy of the vaccine *ϕ*. Over time vaccine protection wears off at a rate *d*, and individuals return to compartment ***S***. We modelled different vaccine efficacies and durations, and proposed different target age groups for vaccination.

### Parameters

We identified the following age groups of interest: 0–4 years old, 5–11 years old, 12–18 years old, 19–24 years old and 25+ years old. These groups were chosen according to the UK education system and represent the preschool years, primary school, secondary school, tertiary education and later years, respectively. EBV seroprevalence was estimated in 12–18 and 19–24-year-olds using stored blood samples from the Health Survey for England (HSE)^16^, an annual cross-sectional survey. The processing of samples from the HSE was approved by the University College London Research Ethics Committee (5683/002). The HSE obtained informed written consent for blood samples to be collected and stored for future analyses. Stratifying by sex and age, 732 samples were randomly selected from individuals aged 11–24 years who participated in the 2002 HSE^4^. Samples were tested for EBV antibody, and the results used to calculate EBV prevalence by age group (Table 1). For younger and older individuals, published studies, were used^3,17–19^.

**Table 1:** EBV prevalence in England, by age group.

Age groups	EBV prevalence
0−4	50%
5−11	55%
12−18	65%
19−24	88%
25+	90%

Natality and mortality data were sourced from the Office for National Statistics (ONS)^20^ for the years 1990 (the mid-point between the birth of the oldest individuals in the HSE sample) to 2016 (the latest year for which demographic data were available) Table 2. We assumed that levels of infections within each age group were at equilibrium, since EBV is not an emerging infection but has co-evolved with humans^21^.

**Table 2:** Table of parameters.

Parameter	Description	Value	Source
Births (*b*)	Number of people born in England between 1990–2016	Data	^20^
Deaths (*μ*)	Natural deaths per age group	Data	^20^
Probability of IM by age (*p*)	Likelihood of progression to IM after EBV infection calculated per age group from IM incidence in Scotland	0.003; 0.040; 0.149; 0.079; 0.843 calibrated from data	^22^
IM recovery rate (*ρ*_*M*_)	Rate of recovery from IM assuming 6 months symptoms duration after clinical onset	2	^32^
Probability of acquiring cancer (*q*)	Likelihood of developing cancer following EBV infection calculated by age group from 2015 ONS cancer data	Data	^20^
Cancer recovery rates (*ρ*_*c*_)	EBV associated cancer recovery rates calculated by fitting an exponential decay to 1 and 5 years survival rate	0.6420 0.8097 0.8895 0.8060 0.5309 fitted from data	^23^
Cancer death rate (*μ*_*c*_)	EBV associated cancer death rates calculated by fitting an exponential decay to 1 and 5 years survival rates	0 0.0964 0.1247 0.0488 0.3819 fitted from data	^23^
Vaccine duration rate (by strategy) (*d*)	Rate of return from “vaccinated” (V) to “susceptible” (S) compartment	0.2; 0.1; 0.05; 0	
Vaccine efficacy (by strategy) (*ϕ*)	Proportion of “vaccinated” compartment (V) protected by vaccine	1; 0.8; 0.6	

The proportions of EBV infections in different age groups that progressed to IM were calculated by the ratio of IM incidence^22^ and EBV incidence. Similarly, the rate of progression to cancer was calculated from data on cancer incidence in England, sourced from the ONS and Cancer Research UK (CRUK)^20,23^. Only the fraction of cases that were EBV-associated were considered^24–26^. Cancer recovery and death rates were calculated by fitting an exponential decay function to 1- and 5-year survival rates^23^. Both IM and cancer rates were calculated from the most recent data available (2012 and 2015, respectively) to ensure our model was up-to-date to inform future vaccine policy. The meanings and sources of the parameters are summarised in Table 2. The model was calibrated to EBV prevalence data by least-squares fitting: the residual distance between model output and data was minimised by varying each age group’s transmission parameter *β*.

Starting from a ’baseline’ vaccine with perfect efficacy and lifelong duration, we reduced both efficacy and duration to study more realistic scenarios. Three different levels of efficacy were tested (60%, 80% and 100%), and four average durations (5 years, 10 years, 20 years and lifelong).

The systems of equations were coded using Matlab2016b. The code used for simulations and producing plots is available on request.

## Results

### Current EBV trends in the absence of a vaccine

**Figure 2:**
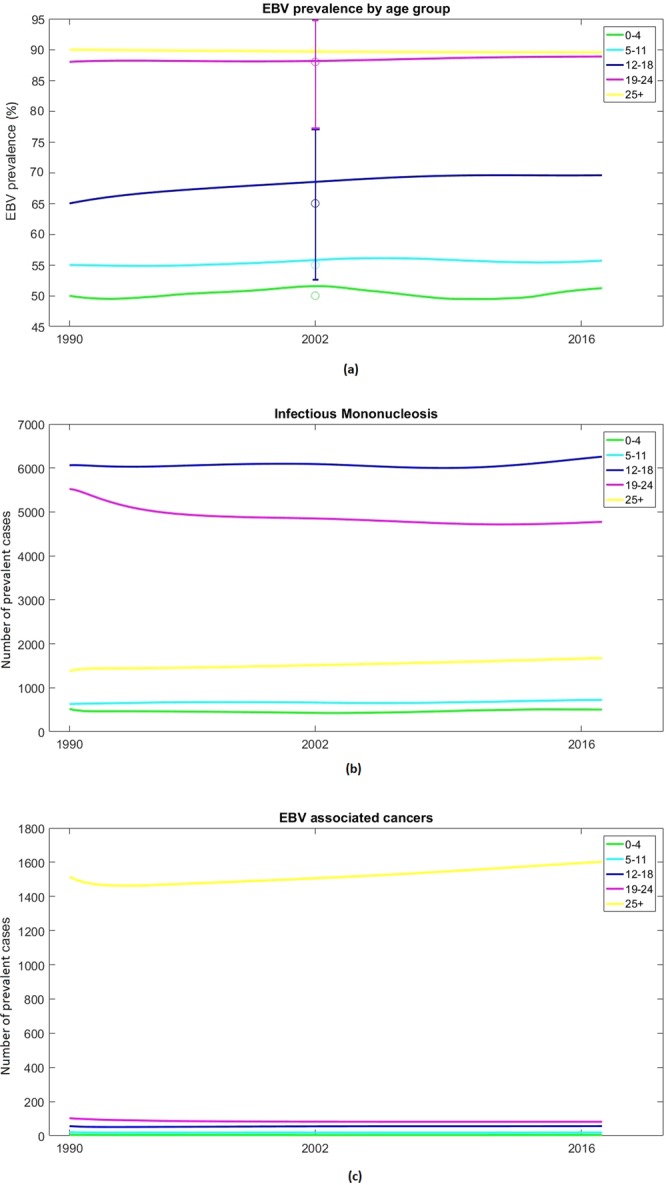
Simulated EBV prevalence, number of prevalent IM cases and EBV-associated cancers, by age, England 1990–2016. The five age groups are 0–4 (green), 5–11 (cyan), 12–18 (blue), 19–24 (pink) and 25+ (yellow). Point markers indicate the EBV prevalence data used for calibration, with 95% confidence intervals shown by the error bars Table 1.

Initially we modelled EBV infection, IM and cancers by age group in the absence of a vaccine. Figure 2a shows simulated EBV prevalence by age group over the 27 years of model running time (1990–2016). Around 50% of individuals were infected before reaching 5 years of age. Seroprevalence then remained relatively stable in the 5–11 age group at ∼55% and increased slightly in the 12–18 age group, to 65–70%. High levels of infection were seen in the 19–24 age group (∼88%) and in over-25s (∼90%).

Figures 2b,c show simulated numbers of IM cases and EBV-associated cancers by age group, respectively. Figure 2b shows that the highest numbers of IM cases are in teenagers (12–18) and young adults (19–24); only a very limited number of cases occur in the youngest age groups. Almost all cancer cases were observed in the over-25s, which fits with the known age distribution of the EBV-associated cancers most commonly found in England.

### Impact of vaccination on EBV infection

**Figure 3:**
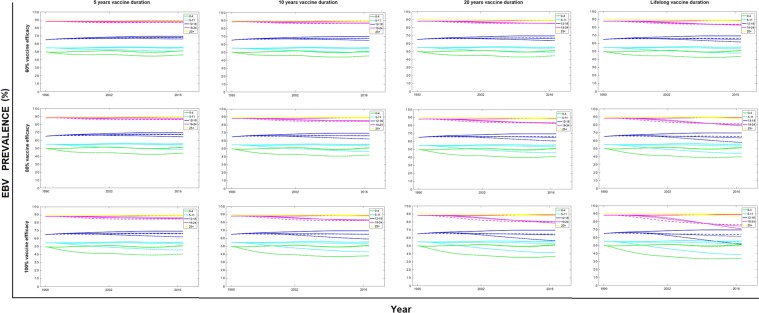
Prevalence of EBV infection, by age group, vaccine duration and efficacy. Results when targeting different age groups are shown in each figure using three dashed lines: (−) when vaccinating individuals in the 0–4 age group, (:) when vaccinating in the 5–11 age group and (−) when vaccinating in the 12–18 age group. These results are compared with EBV prevalence levels when no vaccination is implemented (solid lines).

Having modelled the current epidemiological landscape, we examined the effects of theoretical vaccines and vaccination strategies, starting with the perfect scenario of 100% efficacy and lifelong duration. Figure 3 (bottom-right panel) shows that with a ’perfect’ vaccine, vaccination in the 0–4 age group has the highest overall impact on the whole population, substantially reducing EBV seroprevalence in all age groups.

We then varied vaccine efficacy (60%, 80%, 100%) and vaccine duration (5 years, 10 years, 20 years, lifelong) to investigate the effect of less effective and less durable vaccines. Overall, the impact of vaccine duration on EBV seroprevalence was greater than that of vaccine efficacy. Reducing the efficacy of the vaccine from 100% to 60% limited the success of the vaccine in controlling infection, regardless of the duration of protection. In contrast, at efficacies above 60% the impact of increasing the vaccine duration was greater than improving the efficacy. Specifically, Fig. 3 shows that the difference in the number of EBV infections averted with a vaccine of lifelong duration versus one of 5 years’ duration is considerably higher than the difference in number of infections averted when improving efficacy from 80% to 100%, particularly in older age groups. The choice of the age group to vaccinate is of considerable importance; the younger the age group targeted (0–4 age group), the greater the reduction in EBV seroprevalence observed in the whole population.

### Impact of vaccination on IM disease

**Figure 4:**
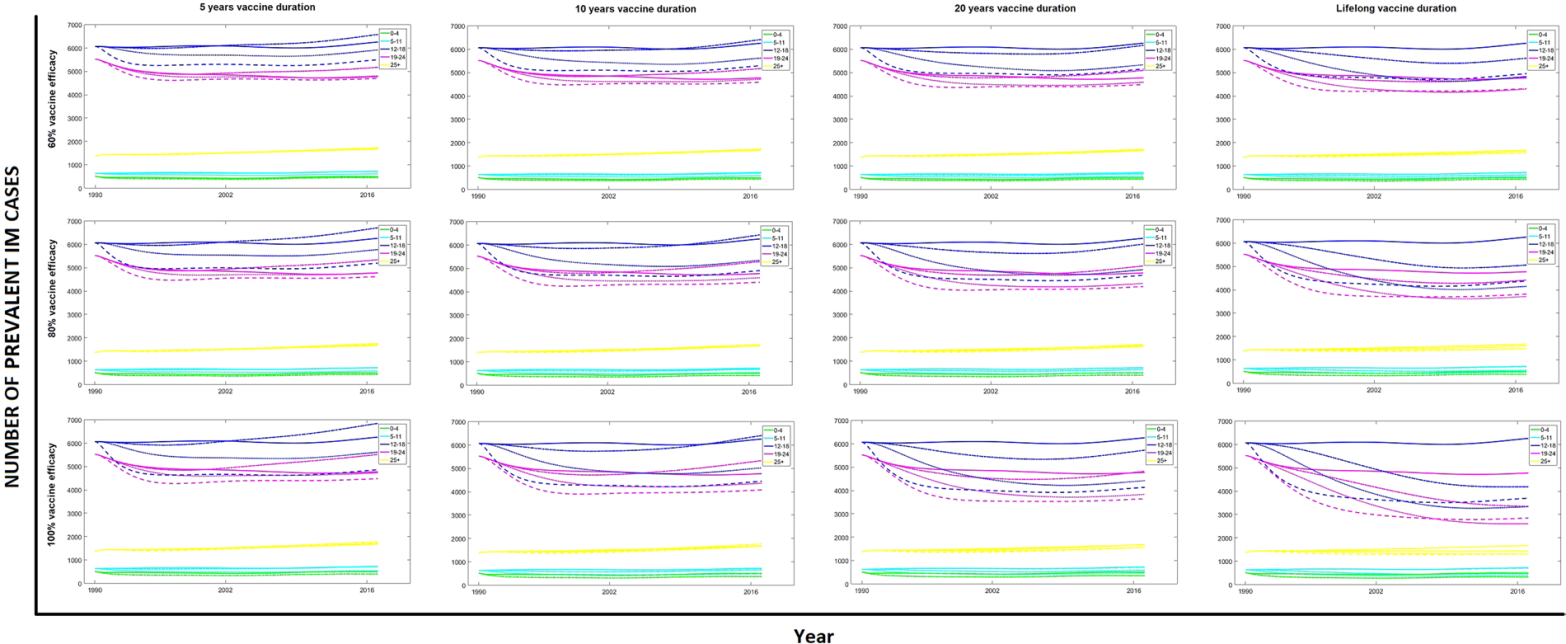
Number of prevalent IM cases, by age group, vaccine duration and efficacy. Results when targeting different age groups are shown in each figure using three dashed lines: (−.) when vaccinating individuals in the 0–4 age group, (:) when vaccinating in the 5–11 age group and (−) when vaccinating in the 12–18 age group. These results are compared with numbers of IM cases when no vaccination is implemented (solid lines).

In the previous section, we highlighted the importance of vaccine duration in reducing EBV prevalence. When analysing the number of prevalent IM cases, this feature is even more important because short duration vaccines, by delaying infection with EBV, could potentially lead to an increase in IM cases (Fig. 4). The ’perfect’ vaccine (Fig. 4; bottom-right panel) has a high impact, reducing the number of IM cases. Its lifelong duration ensures that, even if vaccinated at a young age, individuals have a lifelong protection from disease, especially during the ages when the probability of IM is higher (Fig. 4). This is not the case for less durable and/or less effective vaccines. A combination of high efficacy and short duration of protection creates the most problematic results. The highest efficacies ensure that most vaccinated individuals are protected throughout the duration of the vaccine, but afterwards they may lose protection and become susceptible to EBV at an age when the probability of IM is higher. This outcome depends strongly on the age group targeted. As described above, vaccinating younger individuals, even with an imperfect vaccine (i.e. efficacy <100% and duration less than lifelong) has the greatest impact in reducing EBV prevalence in the population. There is, however, the opposite effect on the numbers of IM cases. If the duration of the vaccine is not long enough, individuals vaccinated in the 0–4 group eventually return to the susceptible compartment during adolescence, when the probability of IM after EBV infection is higher than the age at which they were vaccinated. Consequently, we see a rise of IM cases in older individuals, particularly in the long run. Similar, but less dramatic, results arise when vaccinating in the 5–11 age group (Appendix, Fig. 7), where a vaccine with a duration of at least 10 years would be needed to reduce IM cases in all age groups. The best possible scenarios arise when vaccinating individuals in the 12–18 age group, where IM cases are reduced in any of the scenarios analysed.

### Impact of vaccination on EBV-associated cancers

**Figure 5:**
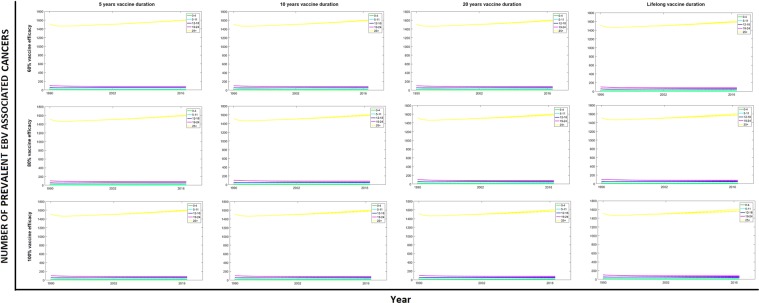
Numbers of prevalent EBV associated cancers, by age group, vaccine duration and efficacy. Results when targeting different age groups are shown in each figure using three dashed lines: (−.) when vaccinating individuals in the 0–4 age group, (:) when vaccinating in the 5–11 age group and (−) when vaccinating in the 12–18 age group. These results are compared with cancer prevalence levels when no vaccination is implemented (solid lines).

**Figure 6:**
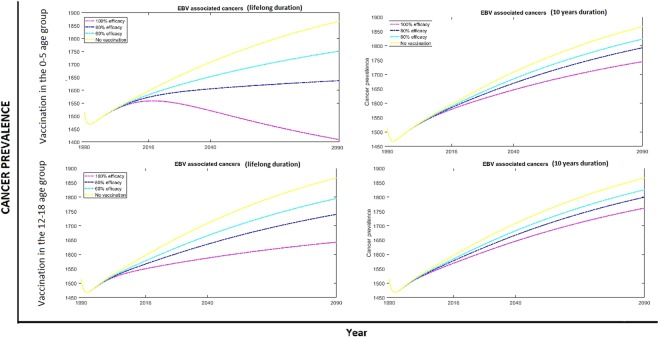
Projected numbers of prevalent EBV associated cancers in the 25+ age group. Lifelong and 10 years vaccine duration were analysed, with varying efficacy of 100% (magenta), 80% (blue) and 60% (cyan), no vaccination (yellow) was also included. Modelling results were projected to 2090 assuming constant average birth and death rates.

Our results show that the impact of EBV vaccination in any of the age groups on related cancers is very small in the short term, even with a perfect vaccine (Fig. 5). This is because the vast majority of cancer cases arise later in life and thus our model does not fully capture them over its 27 year running period. To get a measure of the possible impact of vaccination on EBV-associated cancers in the long run, we projected our modelling results of the number of cancers in the 25+ age group to 2090, assuming constant average birth and death rates (Fig. 6). The increasing number of cancer cases is a consequence of population growth. The scenarios analysed show the importance of vaccine duration in the reduction of cancer prevalence, as both vaccine efficacy and age of vaccination have limited impact when the duration of protection is short. If duration of protection is limited there may be a need for a second dose of vaccine.

Since cancers develop at any point after primary infection, the best strategy in the long run would be the one that offers the maximal reduction of EBV infections i.e. vaccination at 6 months with a long-duration vaccine. If a vaccine has a short duration of protection, vaccinating the oldest age group could be seen as a sensible strategy; however this is ineffective because infection generally occurs before this age.

## Discussion

In this study we created the first compartmental model describing EBV transmission, and calibrated it to data from England. Although EBV infection-preventing (prophylactic) vaccines are not currently available, many are under development and thus studies such as ours have a window of opportunity in which to inform vaccine development and future deployment strategies. In our model, we therefore studied different vaccination strategies (targeting different age groups) in the context of varying both vaccine duration and efficacy.

Our study clearly indicates the importance of the duration of protection offered by future vaccines, which has a higher impact on EBV seroprevalence than vaccine efficacy of the vaccine. Duration of protection should therefore be a core component of the TPP for anti-infection EBV vaccines. Vaccines with poor efficacy (below about 60%) have no significant impact on population prevalence, but for vaccines with better efficacy, a greater reduction in EBV seroprevalence is achieved by lengthening the duration of protection than by further improving efficacy. The duration of vaccine protection also has an important impact on the prevalence of IM disease, as vaccines with a short duration may wear off during adolescence, when the probability of IM is highest. Meanwhile, any type of vaccination has little population-level impact on EBV-associated cancer rates within the time span of our model due to the age distribution of EBV-related cancers in high-income countries such as England. A significant impact of vaccination on cancer rates would only be observable in the long term. In low-income countries where, for example, Burkitt’s lymphoma is more common, we would expect the impact of the vaccine on cancer rates to be more apparent at an earlier stage, as originally proposed by Epstein^27^. Our study was limited by the lack of data to inform some of the model parameters. For example, we do not know the impact on the likelihood of developing IM if infection with EBV is delayed (by vaccination) beyond an individual’s mid-twenties. The rate could remain high, as we see in teenagers in England, or could revert back to that observed in the youngest age groups. Table 3 discusses some areas of uncertainty and proposes potential study designs. Future modelling work could use a Bayesian evidence synthesis approach, combining the mathematical model we present here with data from several study designs, to obtain better parameter estimates and predictions comparing vaccination strategies.

**Table 3:** Areas of uncertainty and potential study designs.

Area of uncertainty	Explanation	Potential study design
Granular EBV prevalence data by age	EBV prevalence data is limited and often outdated.	Cross-sectional seroprevalence study including participants of all ages.
Likelihood of EBV transmission following contact between susceptible and infectious individuals	Infected individuals are not constantly and uniformly infectious. Seasonality and virus shedding mechanisms play a role in the transmission.	Virus shedding analysis could inform individual-level models and give important information on virus transmission.
Likelihood of progressing to infectious mononucleosis if infection by EBV is delayed	Although the likelihood of progression to IM is reasonably well documented for all age groups, it is unknown what will happen if age at infection is delayed due to vaccination.	Prospective cohort study of newly infected individuals in older age groups. Due to the high prevalence of EBV globally this may only be feasible once an infection-preventing vaccine is released.
Cost-effectiveness of different vaccination strategies	Economic evaluation studies are essential to analyse the feasibility of a vaccination program in real settings.	Cost-effectiveness study to calculate threshold values for the cost of EBV vaccine for different vaccination strategies.

Varicella-zoster virus (VZV), currently the only herpes virus with a licensed vaccine, provides some clues as to the characteristics of potential future EBV vaccine. One dose of VZV vaccine is 85% effective at preventing any form of varicella, while two doses are 98% effective^14^. The duration of vaccine protection is not well understood; some studies have shown that most children vaccinated against VZV are protected for at least eight (with one dose^28^) to ten (with two doses^29^) years, and if disease presents later they show only mild symptoms. In the UK varicella vaccination is not part of the routine childhood vaccination programme because delaying infection could increase the risk of (severe) disease in adults^30^. By comparison, the VZV vaccine is given to individuals over the age of 70, to prevent reactivation-associated disease (shingles). The bimodal age distribution of VZV-associated disease thus has critical implications. If a reactivation-preventing EBV vaccine was also developed it could potentially be administered to older, already EBV-infected people to reduce virus reactivation which could potentially also reduce the risk of cancer development.

In any setting, from a policy-making perspective, the best possible EBV vaccination strategy would be to vaccinate all babies once they lose their maternal antibody protection (around 6 months old) if the vaccine has good efficacy (≥80%) and long protection (>20 years). This would ensure the greatest reduction of EBV infections, IM cases and cancers in the long run. However, if the vaccine is protective only for a short time, vaccination at 12 years of age would not substantially affect the prevalence of EBV in the population but would reduce the number of IM cases. Another advantage of vaccination at six months old is that all babies could be vaccinated without needing to test for prior infection. Such testing could be required if teenagers were vaccinated, as more than half would already be infected by their twelfth birthday. Such testing would not necessarily preclude the development of a vaccination programme, as demonstrated by the tuberculosis vaccine BCG, where pre-screening was until recently performed prior to vaccination. An alternative would be to simply vaccinate all teenagers without testing for prior EBV infection, although this would clearly increase overall costs of vaccine supply. Both scenarios would need to be considered when making the economic case for vaccination. Further economic evaluation studies are needed to inform the cost of future vaccines in order to select the most cost-effective strategy. Because IM does not cause mortality, it could be argued that it is acceptable to have a higher number of IM cases in order to reduce EBV seroprevalence and, consequently, the incidence of EBV-associated cancer in the population. In that case, vaccinating babies with a vaccine with a shorter duration of protection may still be an optimal strategy. We should also consider, however, that our model does not account for the potential role of IM as a risk factor for cancers such as Hodgkin’s lymphoma^7,31^. This means that, depending on the prevalence of different cancers in the country of interest, it may be more important to reduce the incidence of IM in the population than EBV seroprevalence, from both an epidemiological and an economical point of view. Thus, vaccination at 12 years old could be a feasible strategy if a vaccine with a limited duration was developed. Future work could focus on studying other countries’ EBV epidemiology. By taking into account local EBV seroprevalence, IM incidence, demographic and cancer data, the model can easily be parametrised to different settings to address suitable vaccination strategies.

In summary, we have described the first model of EBV transmission and used it to determine the impact of hypothetical infection-preventing vaccines with different characteristics. Our work, showing that the duration of vaccine protection will be the key determinant of a successful prophylactic EBV vaccine and the need for this characteristic to be included in a TPP, has important implications for three distinct areas of the drug development pathway. First, at the basic research stage, strategies able to elicit durable protection should be prioritised. Second, for efficacy testing where *in vivo* studies and subsequent first-in-human trials should be designed to measure if protection is sustained. Third, for policy makers where durability of vaccine-mediated protection should be a key factor informing the decision whether to implement a potential vaccine for widespread use in the population.

## Supplementary information

Supplementary Information
