# Supplementary material for: Modelling the dynamics of EBV transmission to inform a vaccine target product profile and future vaccination strategy
Source: Sci Rep. 2019 Jun 26;9:9290. doi: 10.1038/s41598-019-45381-y (PMC6594949; doi:10.1038/s41598-019-45381-y)
Supplement: Supplementary file 1 — Supplementary Information [file 41598_2019_45381_MOESM1_ESM.pdf]

Modelling the dynamics of EBV transmission to inform a vaccine target product profile and future vaccination strategy

Supplementary information

Lara Goscé, Joanne R Winter, Graham Taylor, Joanna Lewis, Helen R Stagg

Model without vaccination

Our model without vaccination consists of the following set of differential equations:

$$\begin{aligned} \dot{S}_i &= a_{1,i} - (\lambda_i + \mu_i)S_i \\ \dot{I}_i &= a_{2,i} + (1-p_i)\lambda S_i + \rho_m D_{IM_i} + \rho_c D_{C_i} - (q_i + \mu_i)I_i \\ \dot{D}_{IM_i} &= a_{3,i} + p_i \lambda_i S_i - (\rho_m + \mu)D_{IM} \\ \dot{D}_{C_i} &= a_{4,i} + q_i I_i - (\rho_c + \mu_c)D_{C_i} \end{aligned} \tag{1}$$

where $i = 1,\ldots,5$ represent the five age groups. The ageing parameters a_k for $k = 1,\ldots,4$ are defined as

$$a_{k,i} = b_{k,i-1}Y_{i-1}(k) - b_{k,i}Y_i(k) \qquad i = 1,\ldots,5 \tag{2}$$

where $Y_i = (S_i, I_i, D_{IM_i}, D_{C_i})$ and $b_{k,i}Y_i(k)$ is the number of people in compartment $Y_i(k)$ getting older and leaving age group i. Also, $b_{1,0} = b$ and $b_{k,0} = 0$ where b is the birth rate and $k = 2,\ldots,4$.

The transmission rate λ is defined by

$$\lambda_i = \beta_i \sum_{j=1}^{5} C(i,j)[I_j + \gamma D_{IM_j} + D_{C_j}] \qquad i = 1,\ldots,5 \tag{3}$$

where β is the infection rate, C is the contact matrix and γ is a parameter that represents the higher infectiousness of individuals with IM that, for lack of data, is currently set equal to 1.

All parameters are defined in Table 2.

Model with vaccination

The model with vaccination consists of the following differential equations:

$$\begin{aligned} \dot{S}_i &= a_{1,i} + dV_i - (\lambda_i + \nu_i + \mu_i)S_i \\ \dot{V}_i &= a_{2,i} + \nu_i S_i - (\tilde{\lambda}_i + d + \mu)V \\ \dot{I}_i &= a_{3,i} + (1-p_i)\lambda_i S_i + (1-p_i)\tilde{\lambda}_i V_i + \rho_m D_{IM_i} + \rho_c D_{C_i} - (q_i + \mu_i)I_i \\ \dot{D}_{IM_i} &= a_{4,i} + p_i \lambda_i S_i + p_i \tilde{\lambda}_i V_i - (\rho_m + \mu_i)D_{IM_i} \\ \dot{D}_{C_i} &= a_{5,i} + q_i I_i - (\rho_c + \mu_c)D_{C_i} \end{aligned} \tag{4}$$

where $i = 1,\ldots,5$ represent the five age groups.

We define the transmission rate to vaccinated individuals $\tilde{\lambda}$ as

$$\tilde{\lambda}_i = (1-\phi)\lambda_i \qquad i = 1,\ldots,5 \tag{5}$$

where ϕ is the vaccine efficacy.

All parameters are defined, and their values are reported, in Table 2.

Additional Figures

In this section we present additional figures showing prevalence of EBV, IM and cancer when vaccinating babies (Fig.2,6,10), children (Fig.3,7,10), adolescents (Fig.4,8,10), young adults (Fig.5,9,10) and adults (Fig.1).

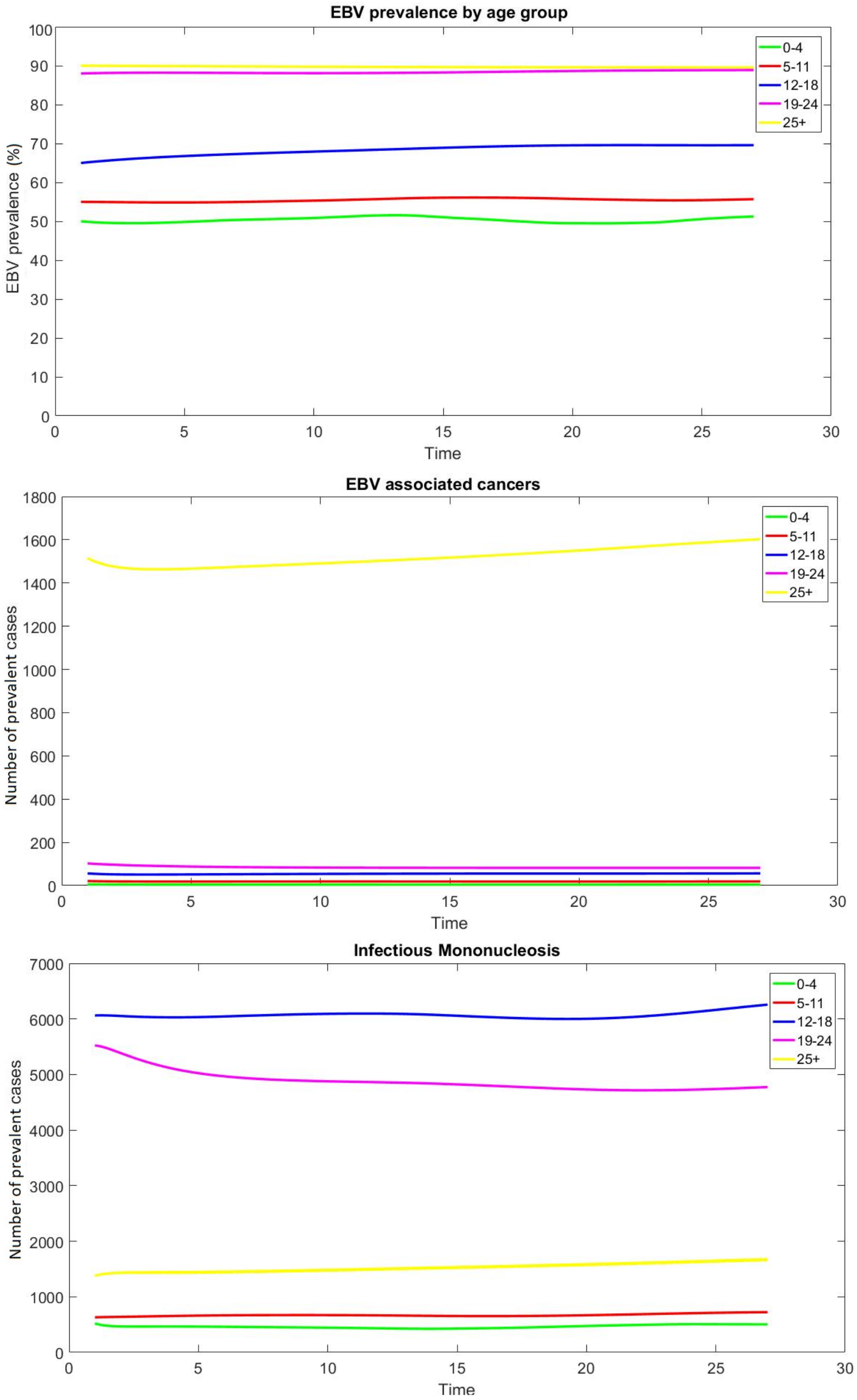

Figure 1. Numbers of EBV, IM and cancer cases following EBV vaccination in the adult population, 25+ age group. Results show that even a perfect vaccine of 100% efficacy and lifelong duration has no effect on the population because only a small number of individuals get infected after 25 years of age.

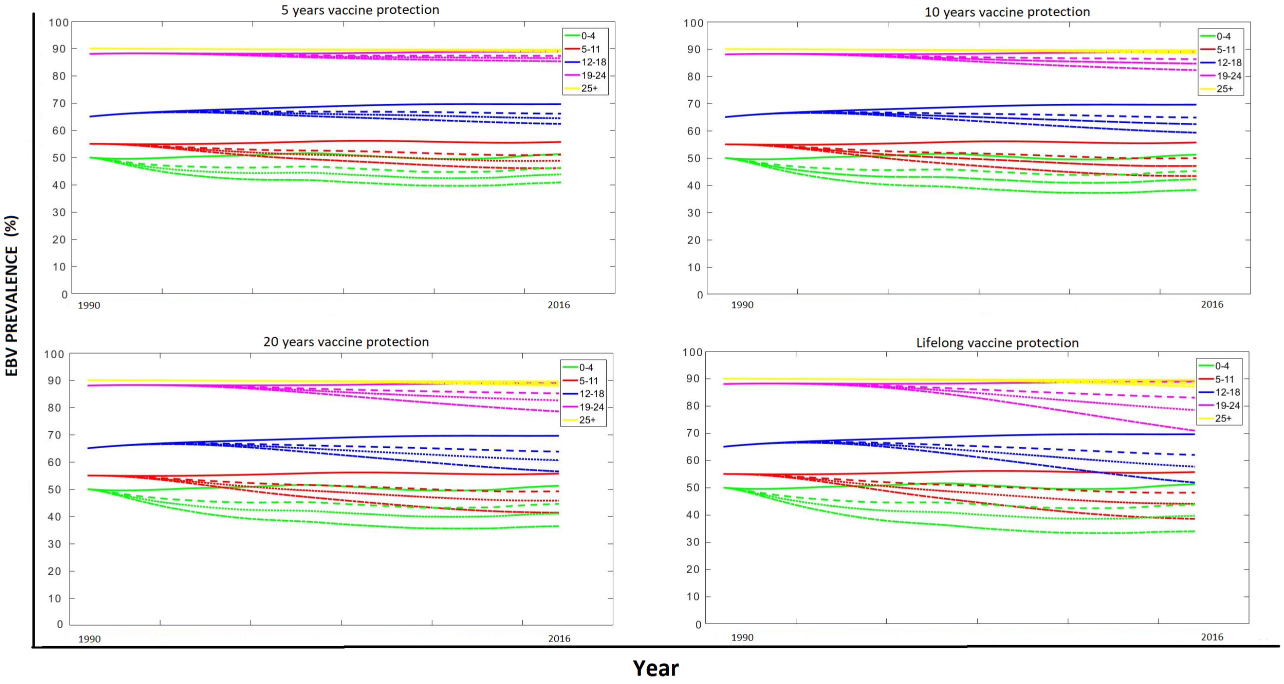

Figure 2. Numbers of EBV cases when vaccinating the 0-4 age group. Results are shown for four different vaccine duration: 5 years (up left figure), 10 years (up right figure), 20 years (bottom left) and lifelong (bottom right). Results for different vaccine efficacy are illustrated using three dashed lines: 60% efficacy (- -), 80% (:) and 100% (-.). The five age groups are 0-4 (green), 5-11 (red), 12-18 (blue), 19-24 (pink) and 25+ (yellow). Results show that vaccinating the 0-4 age group has high effect in all age groups, particularly in the long run.

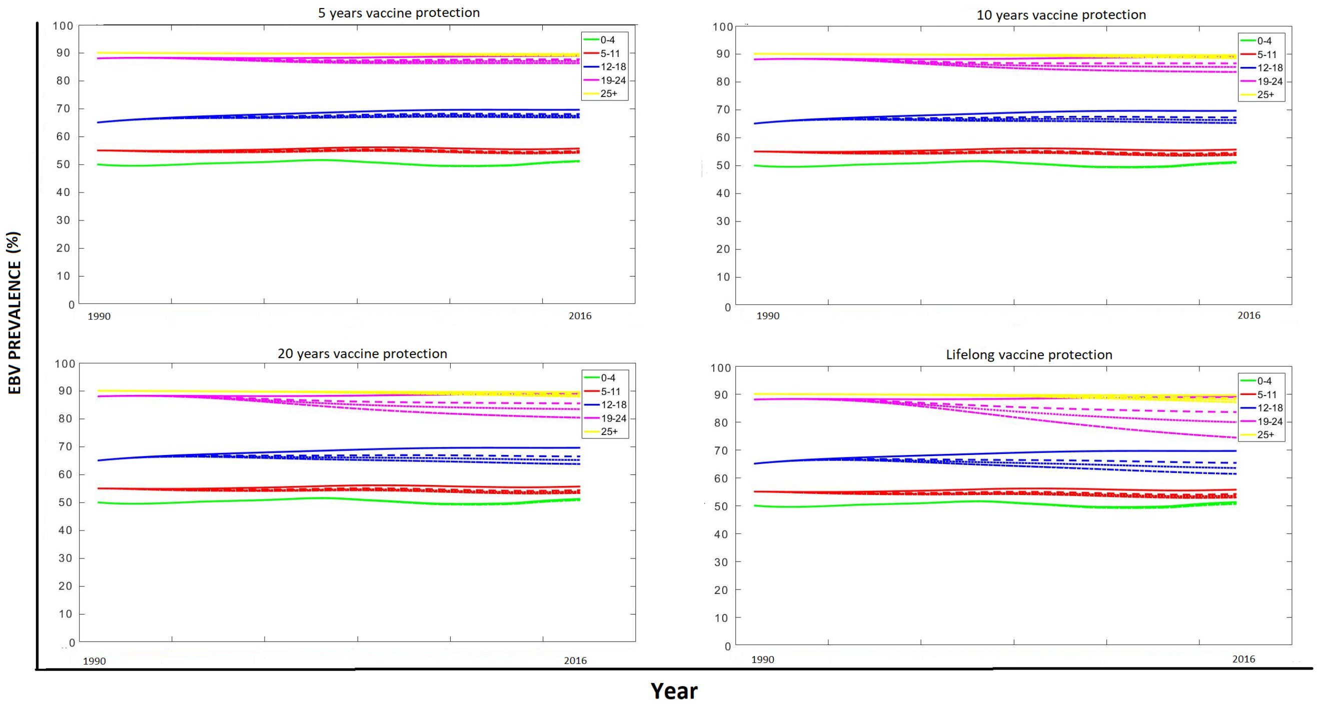

Figure 3. Numbers of EBV cases when vaccinating the 5-11 age group. Results are shown for four different vaccine duration: 5 years (up left figure), 10 years (up right figure), 20 years (bottom left) and lifelong (bottom right). Results for different vaccine efficacy are illustrated using three dashed lines: 60% efficacy (- -), 80% (:) and 100% (-.). The five age groups are 0-4 (green), 5-11 (red), 12-18 (blue), 19-24 (pink) and 25+ (yellow). Results show that vaccinating the 5-11 age group has very small impact, particularly in the younger population.

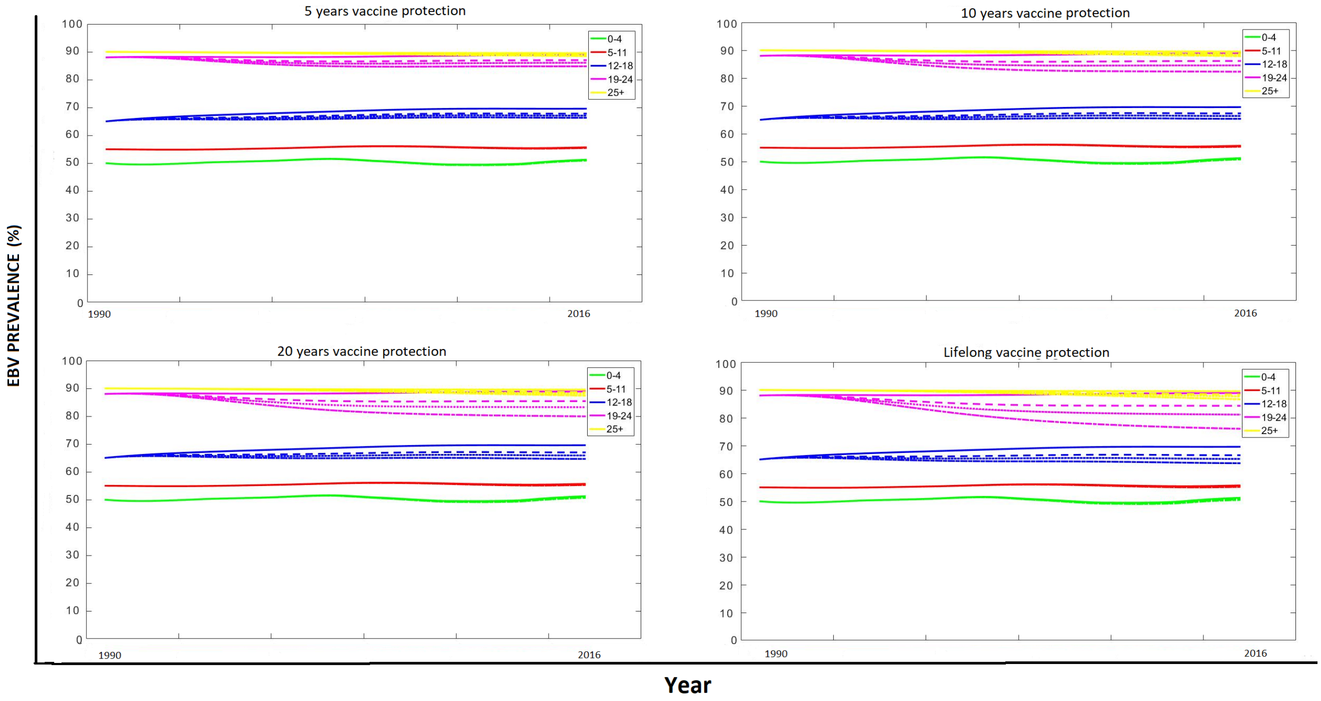

Figure 4. Numbers of EBV cases when vaccinating the 12-18 age group. Results are shown for four different vaccine duration: 5 years (up left figure), 10 years (up right figure), 20 years (bottom left) and lifelong (bottom right). Results for different vaccine efficacy are illustrated using three dashed lines: 60% efficacy (- -), 80% (:) and 100% (-.). The five age groups are 0-4 (green), 5-11 (red), 12-18 (blue), 19-24 (pink) and 25+ (yellow). Results show that vaccinating the 12-18 age group has very small impact, particularly in the younger population.

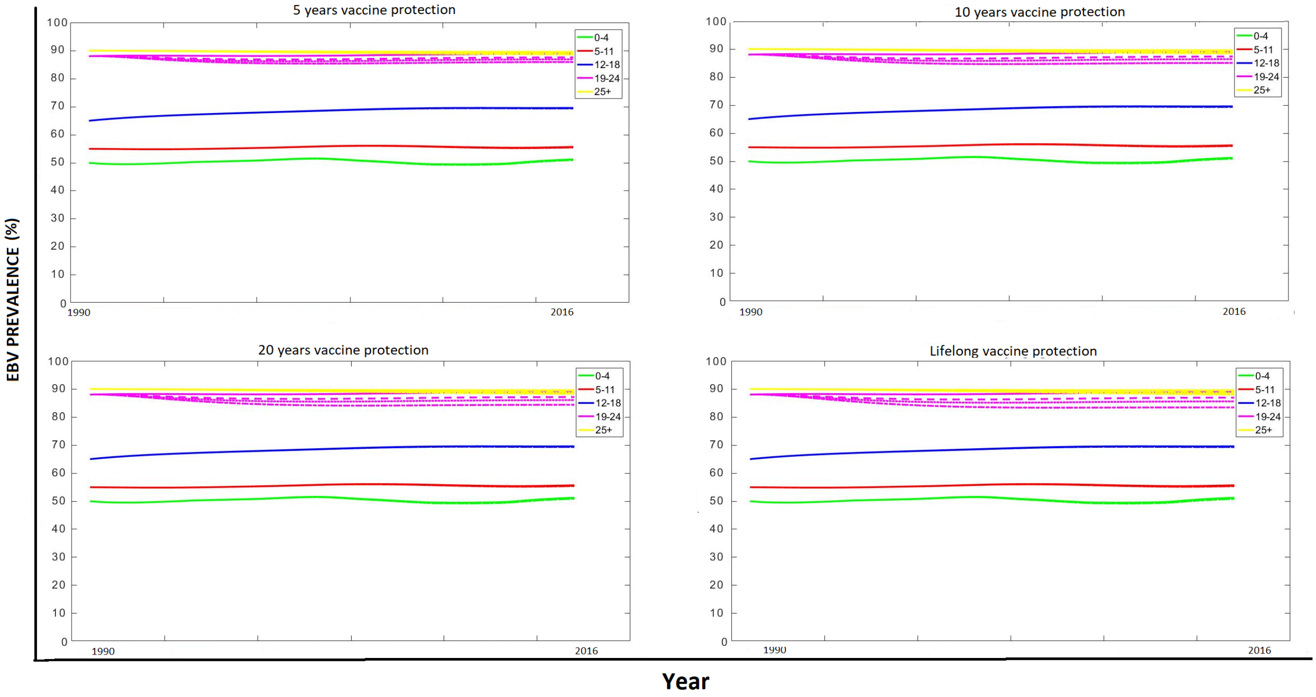

Figure 5. Numbers of EBV cases when vaccinating the 19-25 age group. Results are shown for four different vaccine duration: 5 years (up left figure), 10 years (up right figure), 20 years (bottom left) and lifelong (bottom right). Results for different vaccine efficacy are illustrated using three dashed lines: 60% efficacy (- -), 80% (:) and 100% (-.). The five age groups are 0-4 (green), 5-11 (red), 12-18 (blue), 19-24 (pink) and 25+ (yellow). Results show that vaccinating the 19-25 age group has very small impact in the whole population, to the point that even a longer vaccine duration has little effect.

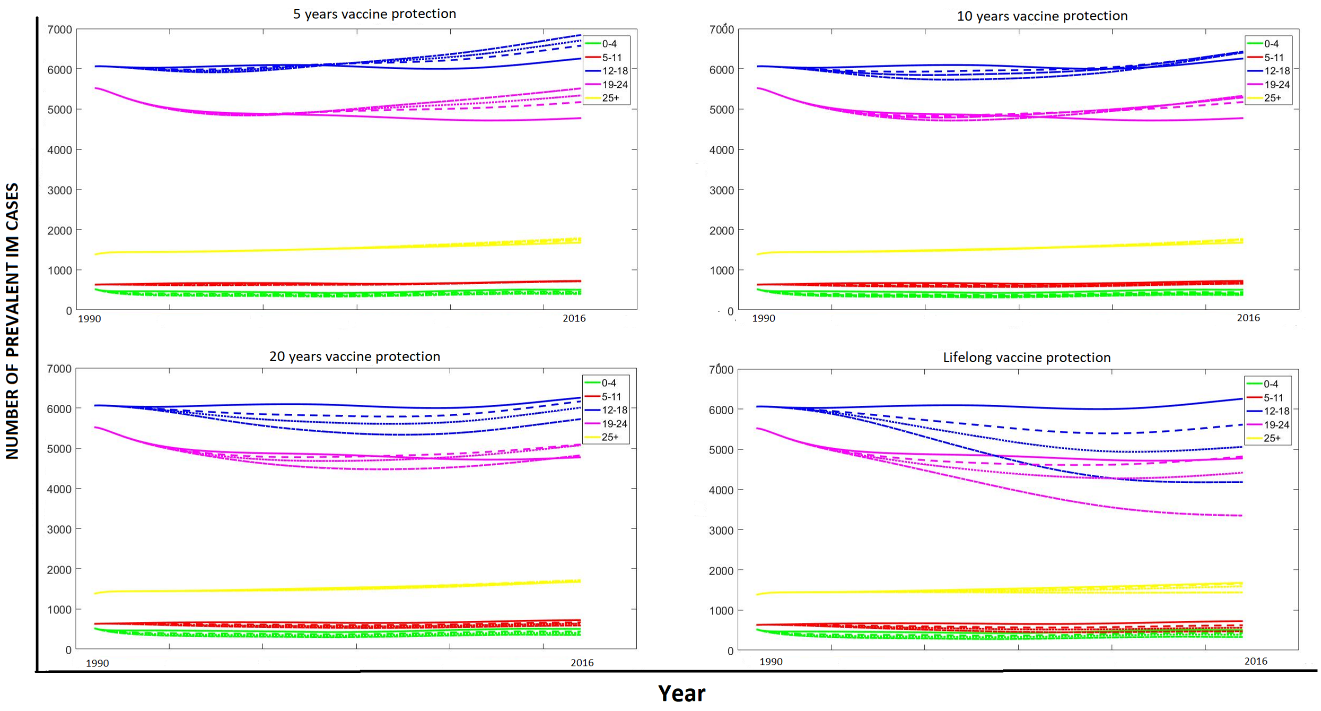

Figure 6. Numbers of IM cases when vaccinating the 0-4 age group. Results are shown for four different vaccine duration: 5 years (up left figure), 10 years (up right figure), 20 years (bottom left) and lifelong (bottom right). Results for different vaccine efficacy are illustrated using three dashed lines: 60% efficacy (- -), 80% (:) and 100% (-.). The five age groups are 0-4 (green), 5-11 (red), 12-18 (blue), 19-24 (pink) and 25+ (yellow). Results show that vaccinating the 0-4 age group has a potentially negative effect on the levels of IM cases in the population if the vaccine has a short or medium duration, particularly in the long run.

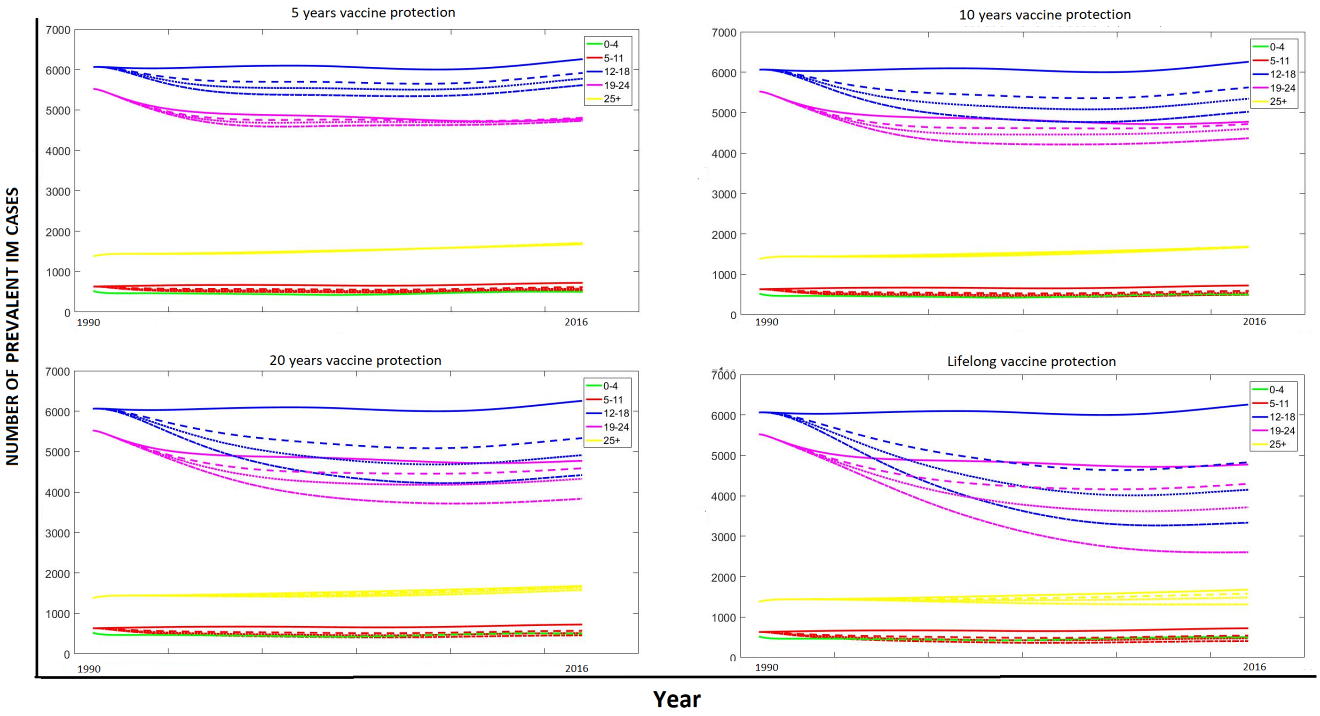

Figure 7. Numbers of IM cases when vaccinating the 5-11 age group. Results are shown for four different vaccine duration: 5 years (up left figure), 10 years (up right figure), 20 years (bottom left) and lifelong (bottom right). Results for different vaccine efficacy are illustrated using three dashed lines: 60% efficacy (- -), 80% (:) and 100% (-.). The five age groups are 0-4 (green), 5-11 (red), 12-18 (blue), 19-24 (pink) and 25+ (yellow). Results show that vaccinating the 5-11 age group has a potentially negative effect on the levels of IM cases in the population if the vaccine has a short duration, particularly in the long run.

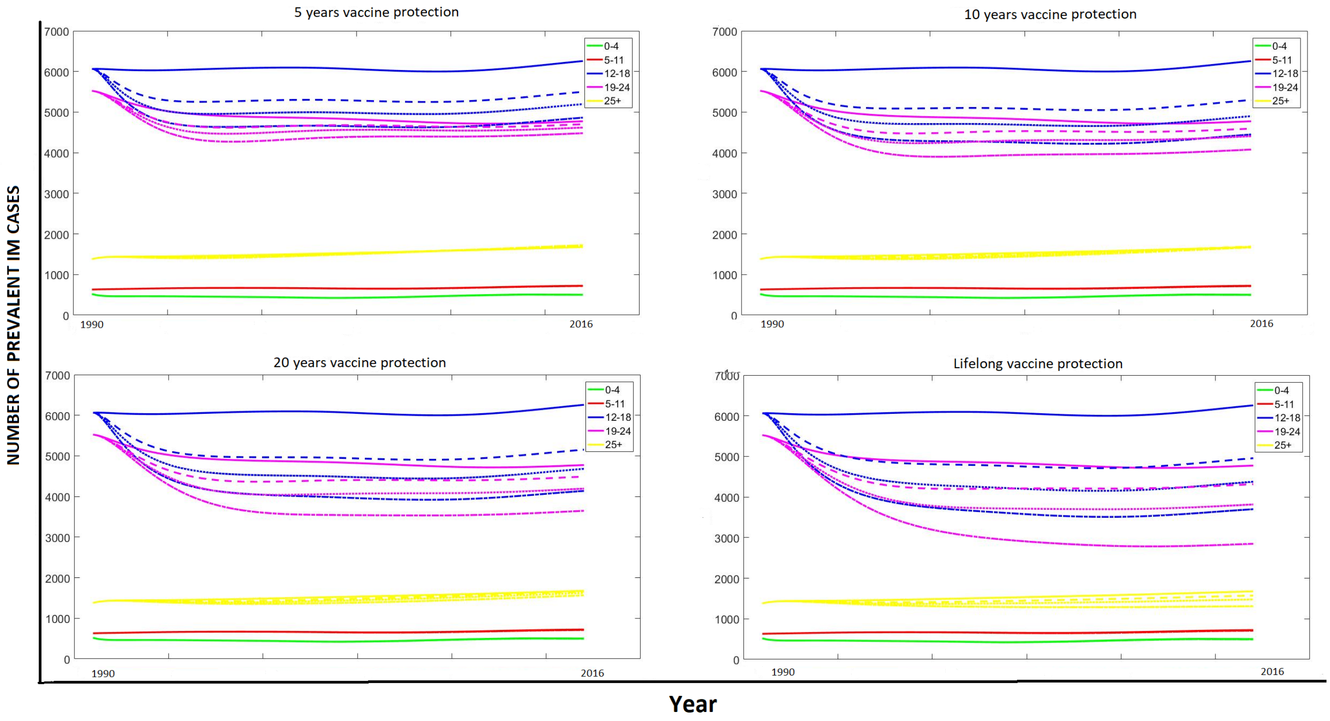

Figure 8. Numbers of IM cases when vaccinating the 12-18 age group. Results are shown for four different vaccine duration: 5 years (up left figure), 10 years (up right figure), 20 years (bottom left) and lifelong (bottom right). Results for different vaccine efficacy are illustrated using three dashed lines: 60% efficacy (- -), 80% (:) and 100% (-.). The five age groups are 0-4 (green), 5-11 (red), 12-18 (blue), 19-24 (pink) and 25+ (yellow). Results show that vaccinating the 12-18 age group has the highest possible impact on the levels of IM cases in the population for any vaccine duration.

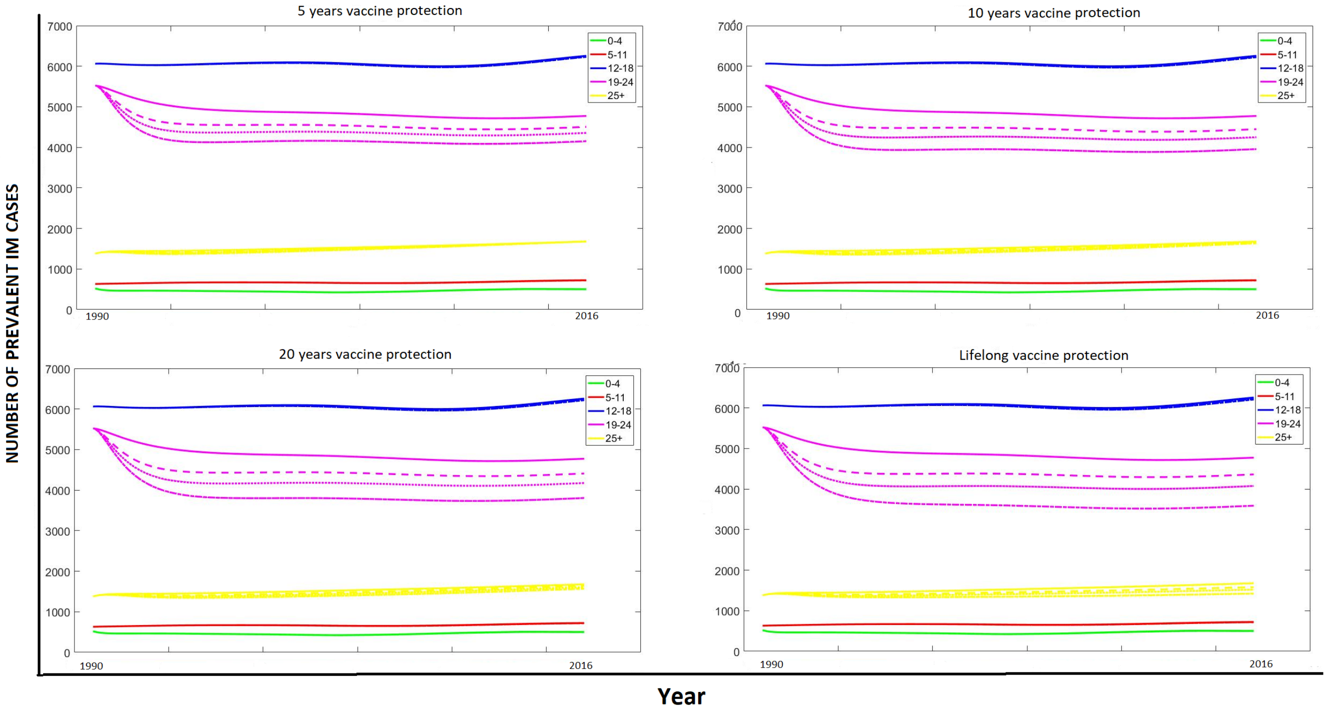

Figure 9. Numbers of IM cases when vaccinating the 5-11 age group. Results are shown for four different vaccine duration: 5 years (up left figure), 10 years (up right figure), 20 years (bottom left) and lifelong (bottom right). Results for different vaccine efficacy are illustrated using three dashed lines: 60% efficacy (- -), 80% (:) and 100% (-.). The five age groups are 0-4 (green), 5-11 (red), 12-18 (blue), 19-24 (pink) and 25+ (yellow). Results show that vaccinating the 19-24 age group has small impact because it does not affect the 12-18 age groups who has the highest number of IM cases.

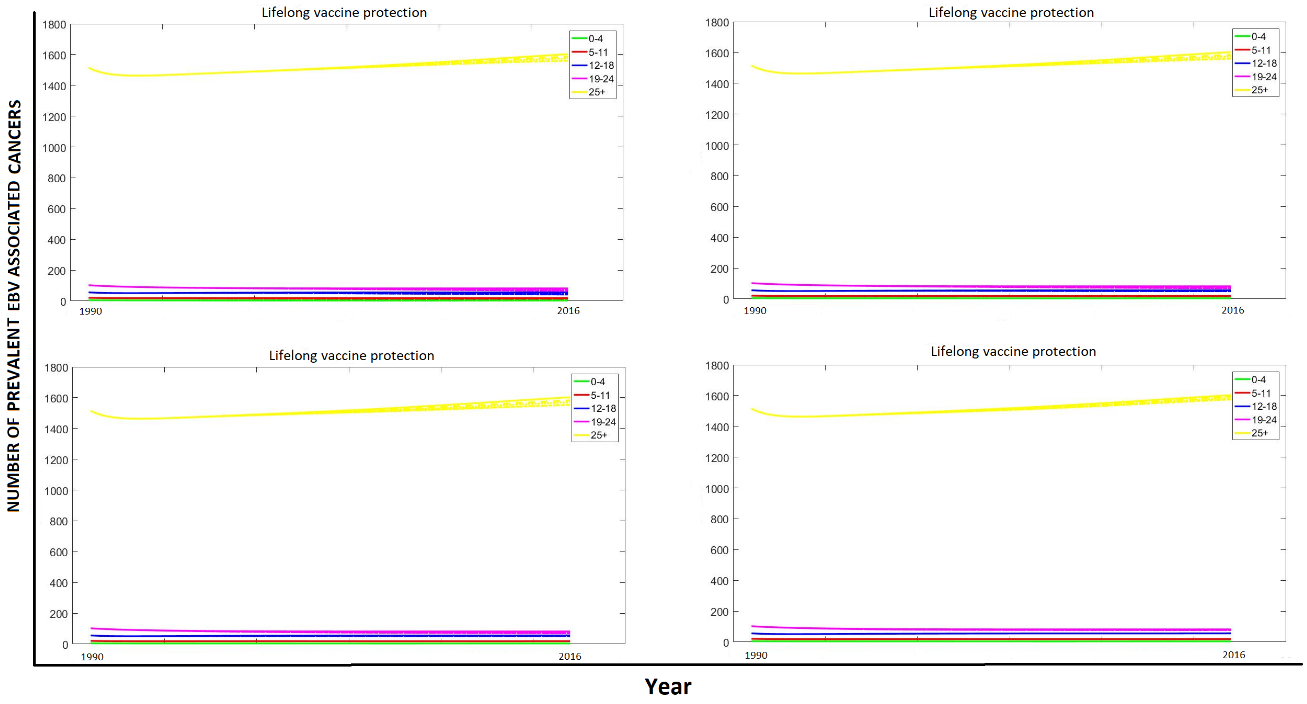

Figure 10. Numbers of EBV associated cancers when using a lifelong vaccine in the 0-4 age group (top left), in the 5-11 age group (top right), 12-18 age group (bottom left) and 19-24 age group (bottom right). Results for different vaccine efficacy are illustrated using three dashed lines: 60% efficacy (- -), 80% (:) and 100% (-.). Results will be clearly visible only when currently infected individuals leave the population however, since cancers develop at any point after primary infection through reactivation, the best strategy in the long run would be the one that offers the maximal reduction of EBV infections i.e. vaccination at 6 months.
